# Ultra-large non-volatile modulation of magnetic moments in PbZr_0.2_Ti_0.8_O_3_/MgO/La_0.7_Sr_0.3_MnO_3_ heterostructure at room temperature via interfacial polarization mediation

**DOI:** 10.1038/s41598-017-03019-x

**Published:** 2017-06-01

**Authors:** Q. Liu, J. Miao, Robert Reeve, K. K. Meng, X. G. Xu, Y. Wu, Y. Jiang

**Affiliations:** 1 School of Materials Science and Engineering, University of Science and Technology Beijing, Beijing, 100083 China; 2 Institut für Physik, Johannes Gutenberg-Universität Mainz, 55099 Mainz, Germany

## Abstract

Multiferroic hybrid structures PbZr_0.2_Ti_0.8_O_3_ (PZT)/La_0.7_Sr_0.3_MnO_3_ (LSMO) and PZT/MgO/LSMO were epitaxially deposited on (001) Nb:SrTiO_3_ crystals. Crystallinity and ferroelectric domain structures were investigated for the PZT/LSMO heterostructure. Interestingly, relatively high non-volatile magnetoelectric coupling effects were observed in both heterostructures at room temperature. The change of chemical valence for Mn and Ti at the PZT/MgO/LSMO interface may play a dominant role rather than external strain or orbital reconstruction, which lead to a large modulation of the magnetization. Correspondingly, the transport behavior of the PZT/MgO/LSMO heterostructure is investigated to confirm the role of oxygen vacancies motion. Our result indicates that the PZT/MgO/LSMO heterostructure have a promising application for future high-density non-volatile memories.

## Introduction

Multiferroic materials which combine multiple ferroic orders such as ferroelectricity and ferromagnetism, have attracted much interests for their promising applications^1, 2^. Moreover, magnetoelectric (ME) coupling is a key effect to realize magnetoelectric random access memories employing electric writing and magnetic reading^3–5^. However, most of the reported achieved ME effects are weak and volatile, which limited the practical application severely. For example, single-phase magnetoelectrics such as BiFeO_3_, HoMnO_3_, TbMnO_3_, etc., face severe limitations due to the weak intrinsic ME coupling^6–8^. As an alternative, composite multiferroic systems with stronger ME coupling and utilizing a wider range of materials can extend the possible applications^3, 9^. It is known that the mechanisms of ME coupling include strain^10^, exchange bias^11^, charge (carrier) modulation^3^, and orbital reconstruction^12^. Among those approaches, the coupling via charge carrier modulation is particularly effective to achieve a relatively strong and non-volatile ME coupling^13^.

The composite multiferroic heterostructure PbZr_0.2_Ti_0.8_O_3_/La_0.7_Sr_0.3_MnO_3_ is one particular system that has received much attentions^13^. La_0.7_Sr_0.3_MnO_3_ (LSMO) is a half-metallic ferromagnetic material with a high spin polarization of nearly 100% and a high critical temperature Tc = 350 K^14^. PbZr_0.2_Ti_0.8_O_3_ (PZT) is a ferroelectric material with a high remnant polarization (Pr ~ 71.9 μC/cm^2^)^15^ and a high Curie temperature (T_C_ = 320 °C)^16^. The ME coupling between LSMO and the PZT interface is complicated and interesting^10, 12, 17^. However, until now, there still exist disputes concerning the primary mechanisms of ME coupling in the PZT/LSMO heterostructure. Moreover, few investigations have been concerned the conductive mechanisms of PZT/LSMO heterostructures to determine its activation energy.

In this work, the modulation of magnetic moments under a non-volatile polarization field in a PZT/LSMO multiferroic heterostructure were investigated. Interestingly, a strong ME modulation up to 63% was achieved in a PZT/LSMO heterostructure room temperature, which is higher than other reported works^13, 18^. Moreover, the modulation of magnetic moments through the polarization field is a non-volatile effect, which is suitable for memory applications. Chemical state change of Mn and Ti at the interface of PZT/LSMO may play a dominant role as opposed to the extrinsic strain or intrinsic orbital reconstruction. Furthermore, an insulating MgO layer was introduced between the LSMO and PZT as a barrier to suppress the orbital reconstruction between Mn and Ti, which results in a maximum ME modulation of 85% at room temperature. Our result indicated the PZT/MgO/LSMO heterostructure may be a promising candidate in future high-density non-volatile memories.

## Results and Discussion

### Structural Characterization

**Figure 1:**
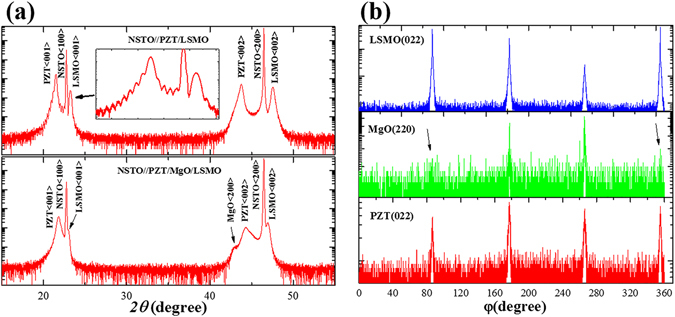
(**a**) XRD *θ-2θ* scans of the NPL and NPML heterostructures respectively. (**b**) Phi-scan for the LSMO, MgO, and PZT layers of the NPML, respectively.

Figure 1 shows the XRD scans of NSTO//PZT(100 nm)/LSMO(20 nm) (NPL) and NSTO//PZT(100 nm)/MgO(5 nm)/LSMO(20 nm) (NPML) heterostructures, respectively. Noted that the thicknesses of the MgO and LSMO layers increased in order to increase the corresponding reflection counts. As shown in Fig. 1(a), only (00 *l*) (*l* is the Miller index) peaks of PZT, MgO and LSMO layers were observed without any impurity phases, indicated all layers were grown with the c-axis normal to the surface of the substrate. An obvious Laue oscillation is shown in the inset of Fig. 1(a), which indicated a high quality of the layers for NPL. Furthermore, since MgO displays a face-centered cubic structure with a lattice constant of 4.216 Å^19^, the PZT unit cell fits well to the corresponding MgO and LSMO unit cells^19, 20^. Figure 1(b) shows XRD φ-scans of PZT (022), MgO (220) and LSMO (022) reflections, respectively. Each layer exhibits an azimuthal diffraction pattern without other peaks in the intervals between four peaks. Accordingly, the fourfold symmetry in the φ-scans reveals the epitaxial nature of PZT, MgO and LSMO layers, indicating a cube-on-cube epitaxial growth on the NSTO substrate.

### Ferroelectric Characterization

**Figure 2:**
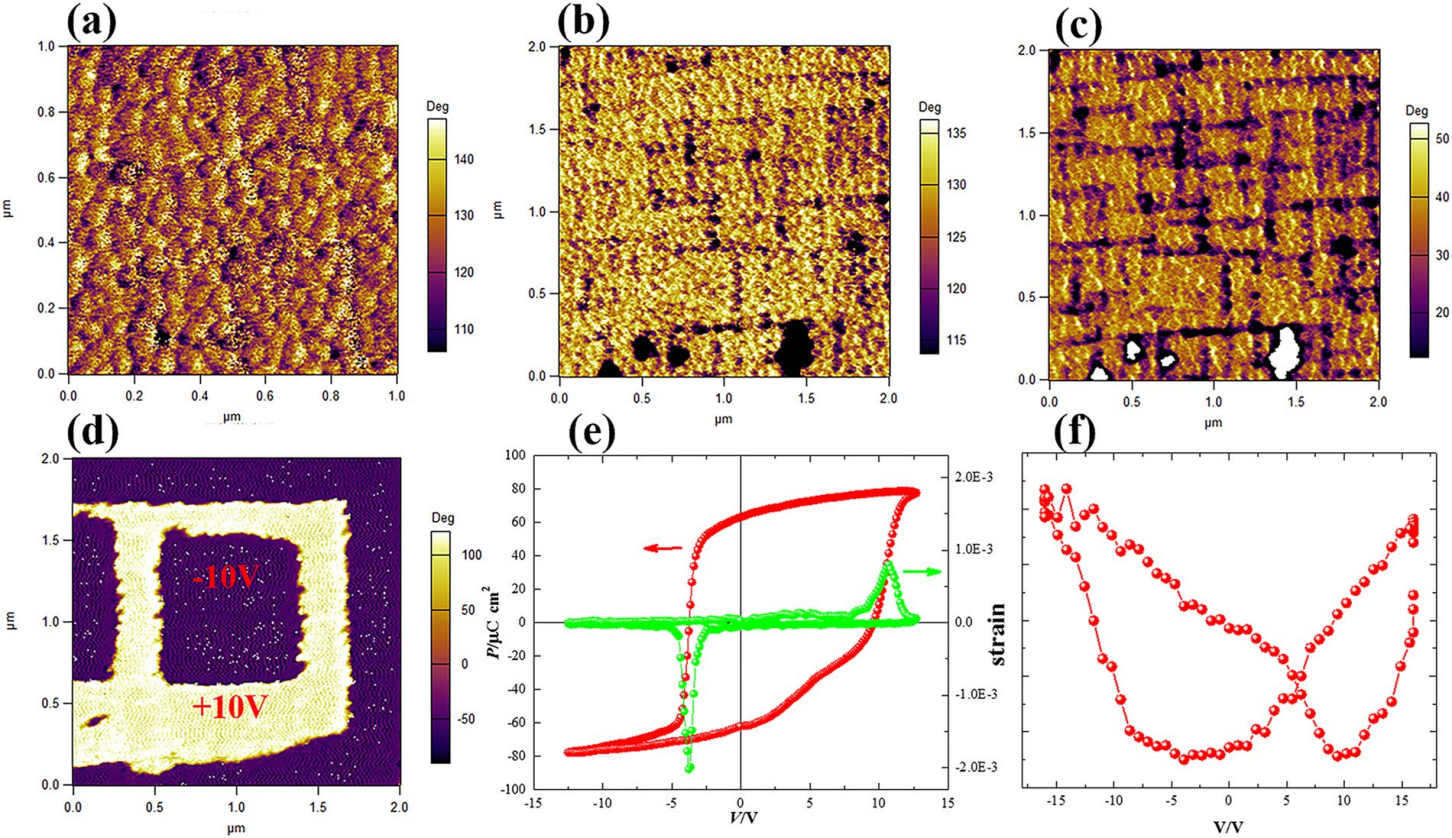
PFM images of the NSTO//PZT (150 nm) structure: (**a**) initial, (**b**) after written under +6 V, (**c**) after written under −6 V and (**d**) after written under ±10 V, respectively. (**e**) Ferroelectric hysteresis and (**f**) strain loop.

Figure 2(a) shows the PFM images of NSTO//PZT (150 nm) before the polarization treatment. The piezoelectric domains and domain-walls in the PZT layer can be seen with the average size of the ferroelectric domains being about 100 nm. The surface roughness R_rms_ of the PZT layer is 0.5 nm which is sufficiently smooth to deposit the LSMO layer. Figure 2(b–d) present the PFM response of NSTO//PZT after writing by +6 V, −6 V and ±10 V, consecutively. The dark and bright regions in the PFM image correspond to the up- and downward- polarization directions (P_up_ and P_down_), respectively. It is noted that the ferroelectric domains of PZT cannot be switched under ±6 V, while the ferroelectric domains can be switched at ±10 V. Moreover, the spontaneous polarization state of NSTO//PZT is downward, which can be attributed to the differences between the work functions of PZT and NSTO^21–23^. Figure 2(e) shows the macro-ferroelectric hysteresis of the NSTO//PZT heterostructure at room temperature. The remnant polarization of NSTO//PZT is estimated to be 63.5 μC/cm^2^, which is consistent with the reported work^13, 22^. Figure 2(f) shows the strain loops of the NSTO//PZT heterostructure under a maximum voltage 15 V. A unidirectional strain response was found in the NSTO//PZT heterostructure instead of a bidirectional one.

### Ferromagnetic Characterization and XPS Analysis

**Figure 3:**
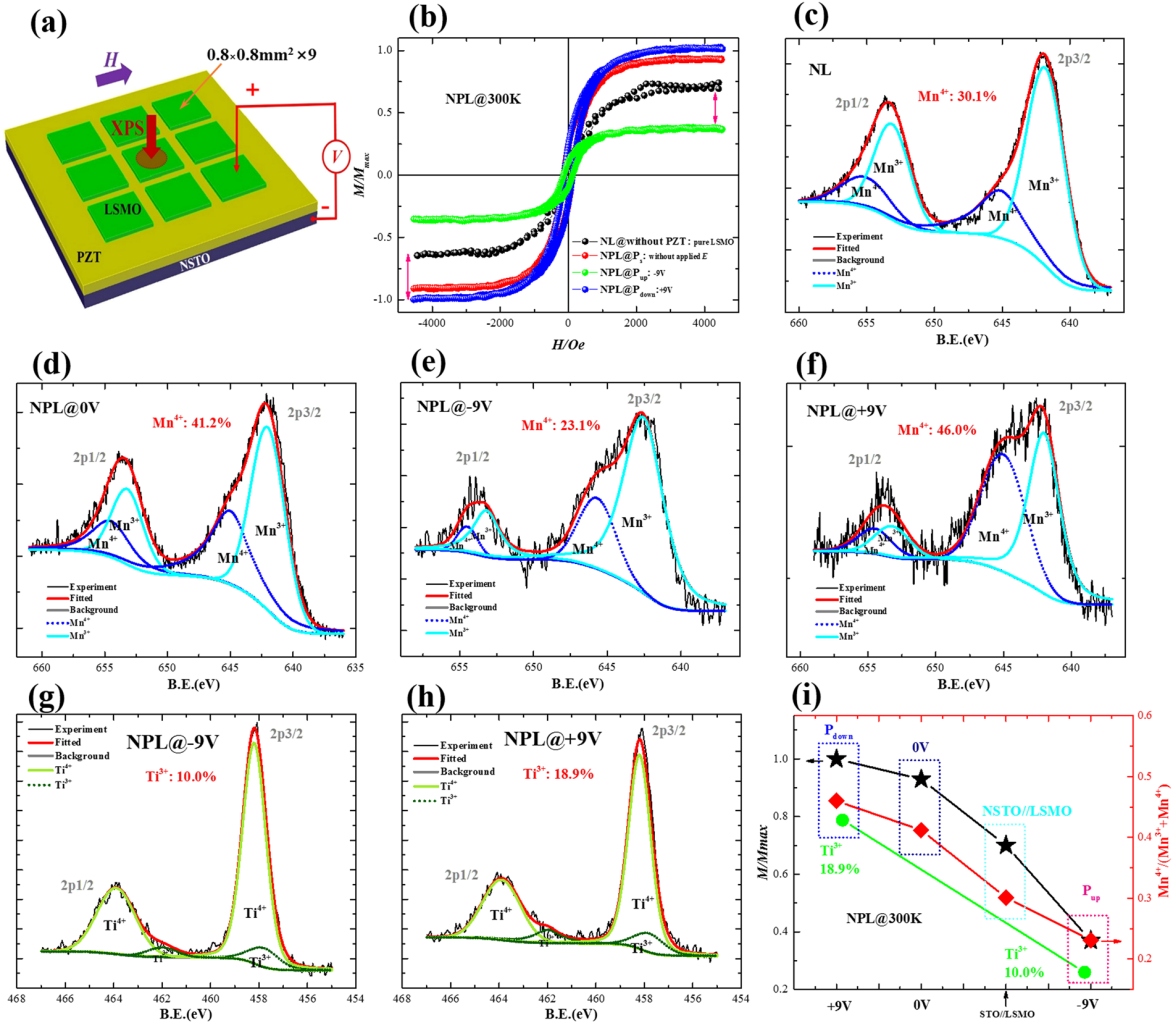
(**a**) Schematic illustration of M-H and XPS measurements, (**b**) M-H curves under various voltages, (**c**–**h**) XPS curves of Mn and Ti ions, (**i**) voltage dependence of Ms and Mn^4+^/Ti^3+^, for the NPL heterostructure.

To investigate the modulation of magnetic moments, the magnetic hysteresis (M-H) and XPS measurements of the NPL heterostructure were performed under different polarization fields at room temperature. As shown in Fig. 3(a), the applied magnetic field of 5000 Oe is parallel to the [010] direction. Before the M-H and XPS measurements, voltages of +9 V or −9 V were applied to fully polarize the ferroelectric PZT layer, and then removed afterwards. As shown in Fig. 3(b), the NPL heterostructure exhibits well-defined hysteresis loops indicating ferromagnetic character at room temperature. Interestingly, the saturated magnetization (Ms) of the NPL heterostructures was changed greatly under different polarization fields of the PZT layer. Compared with pure LSMO (NSTO//LSMO < 4 nm > , abbreviated NL), the Ms of NPL heterostructures increased up to 42.7% [(M_NPL_ − M_NL_)/M_NL_)] under P_down_ and decreased to 47% under P_up_. Assuming this change is due to a change in the effective ferromagnetic thickness with each layer completely modulated and the average magnetic moments of Mn cations doesn’t change, the modulated thickness of LSMO layer is estimated to be 2.5 nm (~6 ML). Accordingly, the relative change of Ms between P_up_ and P_down_, η = [(M(P_up_) − M(P_down_)]/M(P_down_), is estimated to be 63%. Interestingly, the value of η in our NPL sample is higher than other reported works^18^. Moreover, compared with M_NL_ of pure LSMO, the value of Ms of NPL heterostructure at an initial state is increased up to 129%. This phenomenon may be attributed to the downward spontaneous polarization states of the PZT layer^23^. However, the change of Ms between P_down_ and P_up_ for the NPL heterostructure is bidirectional, which is different from the unidirectional strain loops of PZT film as shown in Fig. 2(f). Therefore, the strain effects should not be the primary mechanism of ME coupling in the NPL heterostructure since its bidirectional loops is different from strain loops in PZT film.

To further explore the origin of the ME coupling, XPS analyses were performed to detect the chemical states of Mn and Ti ions at the interface. As shown in Fig. 3(c–f), the chemical states of Mn at the PZT/LSMO interface were changed alongside the change in the magnetization of the heterostructures. The Mn^4+^ content (CMn^4+^) of NL is estimated to be 30.1%, which is consistent with the stoichiometric proportion of LSMO target. However, the content of Mn^4+^ at the PZT/LSMO interface can be estimated to be increased up to 41.2% without electric field, down to 23.1% under P_up_ and further increased to 46.0% under P_down_, respectively. Accordingly, the relative change of Mn^4+^ contents between P_up_ and P_down_ is estimated to be 50%. Moreover, as presented in Eq. (1), Ti^3+^ can be transformed into Ti^4+^ through an oxidation reduction due to oxygen deficiency in the film^24^. Meanwhile, Mn^3+^ and Mn^4+^ ions can be converted through the actions of oxygen vacancies as described by Eq. (2)^24, 25^.

1 $$2{({{{\rm{Ti}}}^{4+}}_{{{\rm{Ti}}}^{4+}})}^{x}+[{({{{\rm{O}}}^{2-}}_{{{\rm{O}}}^{2-}})}^{x}]=2{({{{\rm{Ti}}}^{3+}}_{{{\rm{Ti}}}^{4+}})}^{-}+[{({V}_{{{\rm{O}}}^{2-}})}^{2+}]+\frac{1}{2}{{\rm{O}}}_{2},$$

2 $$2{({{{\rm{Mn}}}^{3+}}_{{{\rm{Mn}}}^{3+}})}^{x}+[{({V}_{{{\rm{O}}}^{2-}})}^{2+}]+\frac{1}{2}{{\rm{O}}}_{2}=2{({{{\rm{Mn}}}^{4+}}_{{{\rm{Mn}}}^{3+}})}^{+}+[{({{{\rm{O}}}^{2-}}_{{{\rm{O}}}^{2-}})}^{x}],$$

At the PZT/LSMO interface, those dynamic process would play a key role in the motions of the oxygen vacancies. Figure 3(g) and (h) present the ratios of Ti^3+^/Ti^4+^ at the PZT/LSMO interface for positive and negative polarizations of the NPL heterostructure, respectively. The ratios of Ti^3+^/Ti^4+^ at the PZT/LSMO interface were estimated to be 10.0% @−9V (P_up_) and 18.9% @+9V (P_down_), respectively. It is known that the magnetization of LSMO can be modulated by the changing of the ratio of Mn^3+^/Mn^4+^. As a result, the total magnetization of NPL heterostructure can be modulated accordingly. As shown in Fig. 3(i), the change of Ms in the NPL heterostructure is consistent with the ratios of Mn^4+^ and Ti^3+^. Thus, the motions of oxygen vacancies at the PZT/LSMO interface due to the polarization field, would play a dominant role in modifying the chemical state of Mn and Ti, resulting in a high modulation of the magnetization.

**Figure 4:**
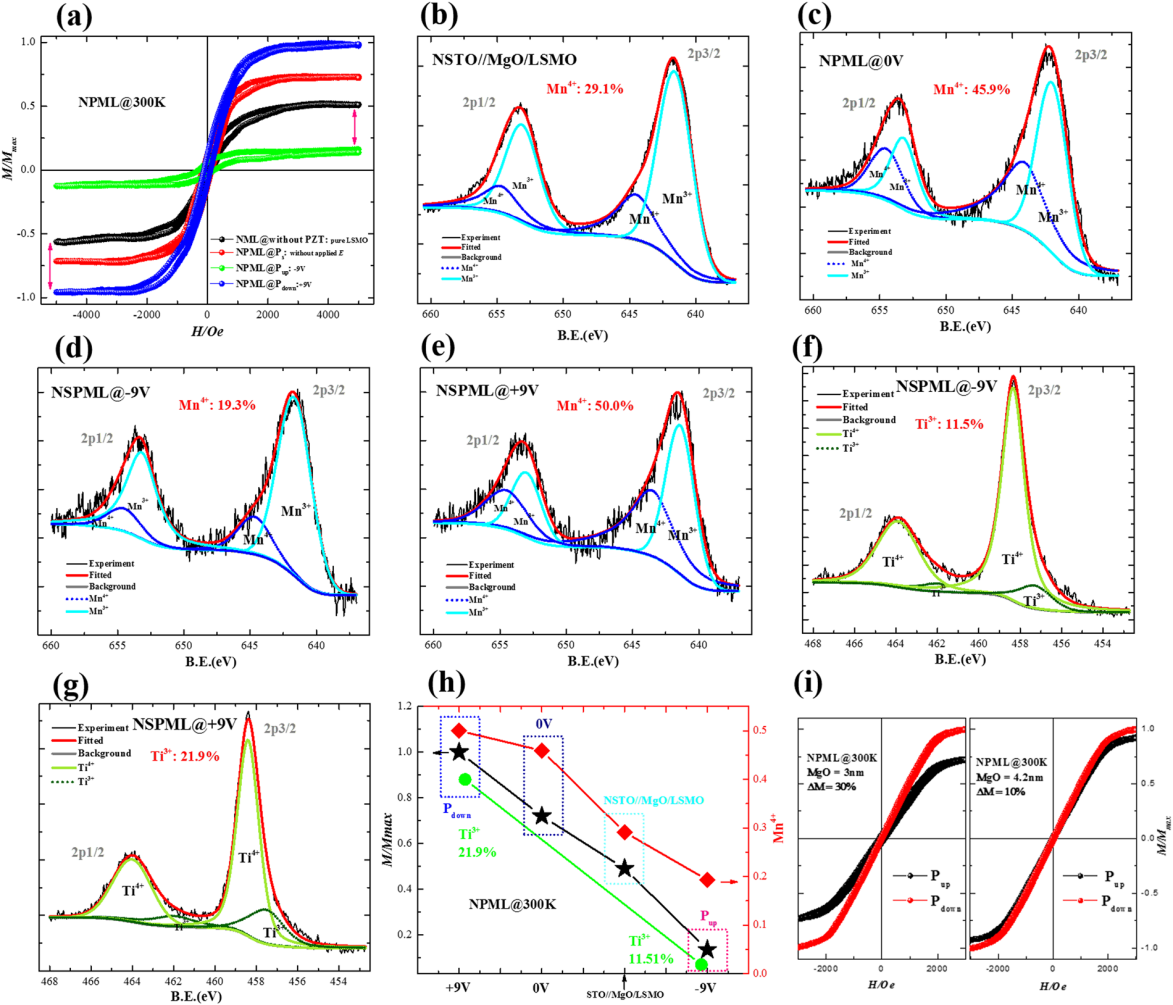
(**a**) M-H curves under various voltages, (**b**–**g**) XPS curves of Mn and Ti ions, (**h**) the dependence of Ms, the ratio of Mn^4+^ and Ti^3+^on voltages, (**i**) M-H curves under various voltages with different thickness of MgO, for the NPML heterostructure.

To exclude the orbital reconstruction effect proposed by Cui *et al*.^12^, an insulating MgO layer was introduced between the LSMO and PZT layers of the NPML heterostructure. Thus, the Ms of NPML heterostructure should be mainly dominated by the MgO/LSMO interface instead of the PZT/MgO interface^26^. As shown in Fig. 4(a), compared with the pure LSMO (NSTO//MgO<2.1 nm>/LSMO<4 nm>, abbreviated NML), the Ms of the NPML heterostructure increased up to 100% for P_down_ and decreased to nearly 70% for P_up_ at room temperature. Thus, the relative change of Ms between P_up_ and P_down_ is 85% (compared to P_down_). The modulation trend of the NPML heterostructure is very similar to that of the NPL heterostructure presented in Fig. 3.

As shown in Fig. 4(b–e), the chemical states of Mn of the NPML heterostructure were detected by XPS under different polarization fields. From XPS analysis, the content of Mn^4+^ in the NPML heterostructure is 19.3% (P_up_), 45.9% (0 V) and 50.0% (P_down_), respectively. Accordingly, the relative change of Mn^4+^ contents between P_up_ and P_down_ is estimated to be 61.4%. Correspondingly, the contents of Ti^3+^ are estimated to be 11.5% (P_up_) and 21.9% (P_down_). Figure 4(h) indicates the trend of Ms^+^ of NPML heterostructure is consistent with the ratios of Mn^4+^ and Ti^3+^. Since the possible orbital reconstruction between Mn and Ti is suppressed by the inserted MgO layer, the higher ME coupling of the NPML heterostructure may be mainly originated from the charge effect rather than orbital reconstruction. Furthermore, it is worth noting the modulation coefficients of ME coupling in NPML (η = 85%) is higher than that in NPL (η = 63%). Since the MgO layer was deposited in an anaerobic atmosphere^27^, then more oxygen vacancies defects would be formed in the PZT film of NPML heterostructure.

MH measurements for NPML heterostructure with different thickness of MgO layer were performed in Fig. 4(i). As shown, the changes of magnetic moments between P_up_ and P_down_ states are about 30% (with 3 nm MgO) and 10% (with 4.2 nm MgO), respectively. Compared with NPML of 2.1 nm MgO layer (~85%), the changes of magnetic moments with thicker MgO layers show a decreasing tendency. A possible reason is that the MgO thickness is thicker than the designed barrier^28^. The ME coupling between LSMO and PZT becomes weaker with the increasing of MgO layer thickness.

### DC Leakage Current Properties

**Figure 5:**
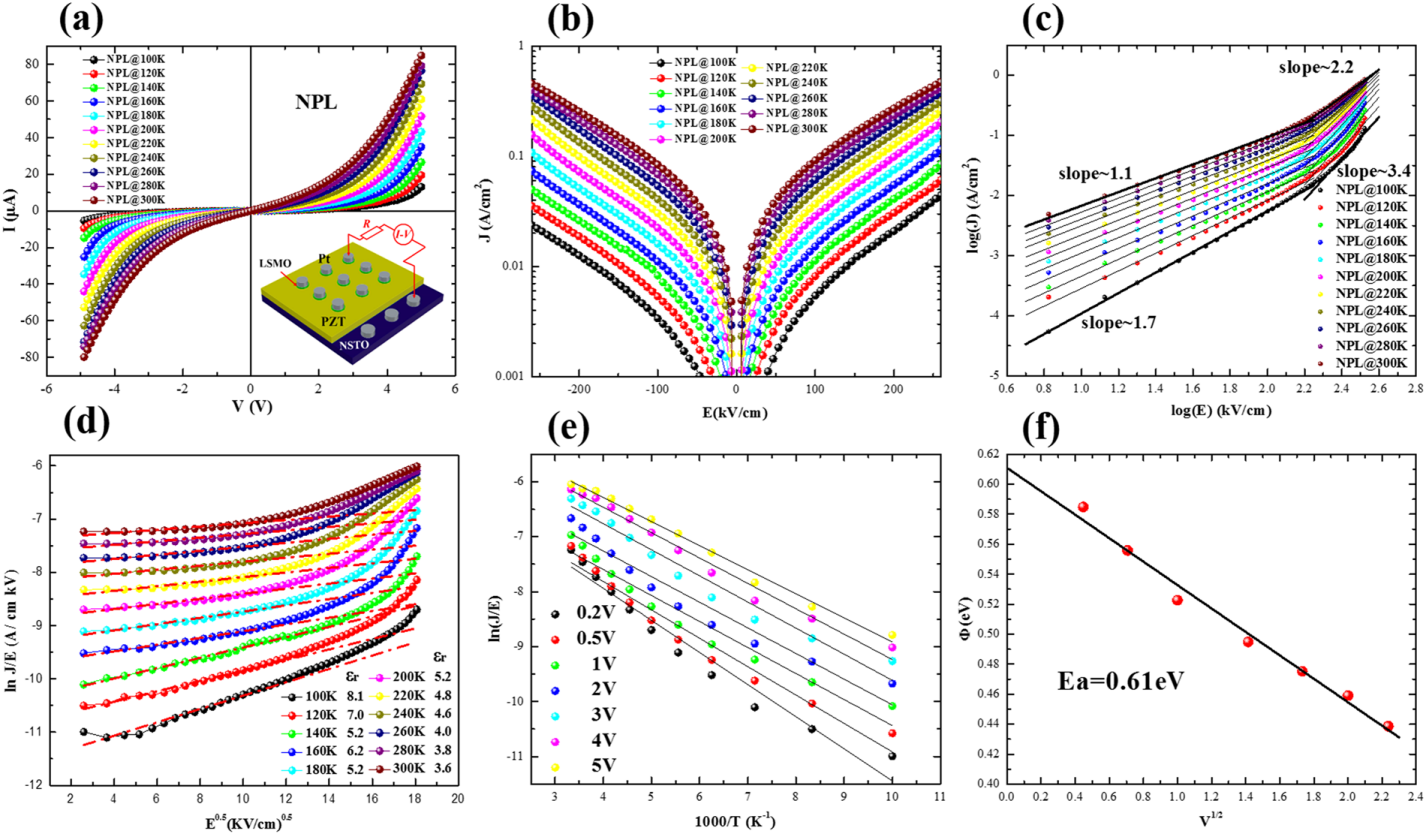
(**a**) Illustration of I-V measurements under different temperatures, (**b**) temperature and voltage dependence of J, (**c**) SCLC analysis at positive region, (**d**) ln(J/E) ~ E^1/2^ at negative region, (**e**) temperature dependence of ln(J/E), and (**f**) relationships between activation energies and voltage for the NPL heterostructure.

Figure 5(a) and (b) present the current-voltage (*I-V* and *J-E*) characteristics of NPL heterostructures measured at various temperatures. The *J-E* characteristics were carried out using a Keithley 2430 pulsemeter, and a staircase-shaped dc bias voltage with a step of 0.05 V and span of 3 sec was used. Figure 5 (c) shows plots of log (J) versus log (E) for positive bias, which according to the space-charge-limited–current (SCLC) model is given as^29^

3 $$J={9}{\varepsilon }_{{0}}{\varepsilon }_{{\boldsymbol{r}}}\mu \theta {V}^{{2}}/{8}{d}^{{3}}$$

where ε_0_ is the permittivity of free space, μ is the charge carrier mobility, d is the thickness of the film, ε_r_ is the low-frequency permittivity of the film and θ is the ratio of the free carriers to the trapped carriers. As shown, under low electric fields and in the high temperature region (>160 K), the fitting slopes of the curves are close to 1, indicating Ohmic behaviour in this region. Moreover, the values of the slopes approach 2 for lower temperatures (<160 K), suggesting a SCLC conduction mechanism in the NPL heterostructure.

Alternatively, the current behavior can also be associated with a Poole–Frenkel mechanism according to^30^

4 $$J=CE\,\exp [\frac{-({\varnothing }_{t}-e\sqrt{eE/\pi \varepsilon {{\epsilon }}_{r}{\varepsilon }_{0}})}{KT}],$$

where *C* is a constant, *K* is the Boltzmann’s constant, *T* is the temperature, *ε*_0_ is the permittivity of vacuum, ∅_*t*_ is the trap ionization energy, *e* is the electron charge and *∈*_*r*_ is the optical dielectric constant. Figure 5(d) shows the ln (J/E) versus E^1/2^ for negative bias and the temperature dependence on permittivities ε_r_. As shown in the figure, the plot exhibits a linear relationship, which is associated with a thermal electron emission to overcome the Coulomb barrier^31^. To determine the value of the trap ionization energy, the relationships between the leakage current and temperature were also determined for the NPL heterostructure. Figure 5(e) shows the plots of ln(J/E) vs 1000/T under fixed voltages. Furthermore, the ionization energies can be straightforwardly extracted from the slopes as shown in Fig. 5(f). As estimated from the slope of the linear fits, the field dependence of the ionization activation energy yields a zero-field trap ionization energy E_t0_ of 0.61 eV. It is known that oxygen vacancies are one of the mobile ionic defects in an oxide film^32^. Specifically, in Pb-based ferroelectrics, the E_t0_ of oxygen vacancies is usually in the range from 0.56 eV to 1.58 eV^33–36^. Thus, the dc conductivity in the NPL heterostructure can be attributed to the electric field induced migration of oxygen vacancies.

**Figure 6:**
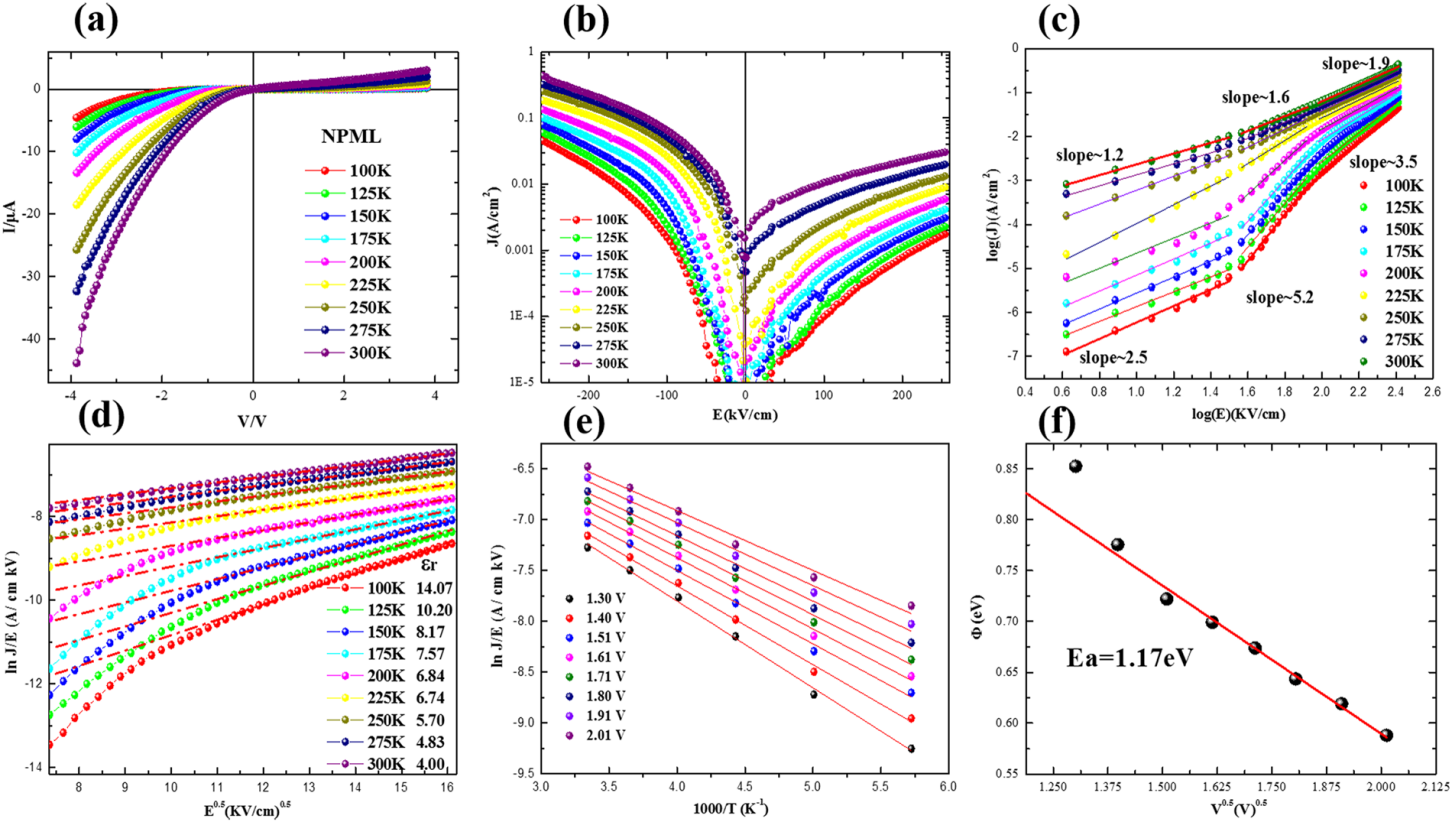
(**a**) Illustration of I-V measurements under different temperatures, (**b**) temperature and voltage dependence of J, (**c**) SCLC analysis at positive region, (**d**) ln(J/E) ~ E^1/2^ at negative region, (**e**) temperature dependence of ln(J/E), and (**f**) relationships between activation energies and voltage for NPML heterostructures.

Figure 6(a) and (b) present the current-voltage (*I-V* and *J-E*) characteristics of NPML heterostructure measured at various temperatures. Figure 6(c) shows plots of log(J) versus log(E) for positive bias, which according to the space-charge-limited–current (SCLC) model^29^. Alternatively, the current behavior can be associated with a Poole–Frenkel mechanism^30^. Figure 6(d) shows the ln(J/E) versus E^1/2^ for negative bias and the temperature dependence on permittivities ε_r_. The linear relationship between ln(J/E) and E^1/2^ indicated a thermal electron emission to overcome the Coulomb barrier^31^. Figure 6(e) shows the plots of ln(J/E) vs 1000/T under fixed voltages. Furthermore, the ionization energies can be straightforwardly extracted from the slopes as shown in Fig. 6(f). As estimated from the slope of the linear fits, the field dependence of the ionization activation energy yields a zero-field trap ionization energy E_t0_ of 1.17 eV. Thus, the dc conductivity in the NPML heterostructure can be attributed to the electric field induced migration of oxygen vacancies^37^. It should be noted that NPML show an obvious asymmetric I-V behavior than that of NPL which may be attributed to the asymmetric structure and changed barrier height by MgO layer^38, 39^.

### Mechanism analysis of magnetization modulation

**Figure 7:**
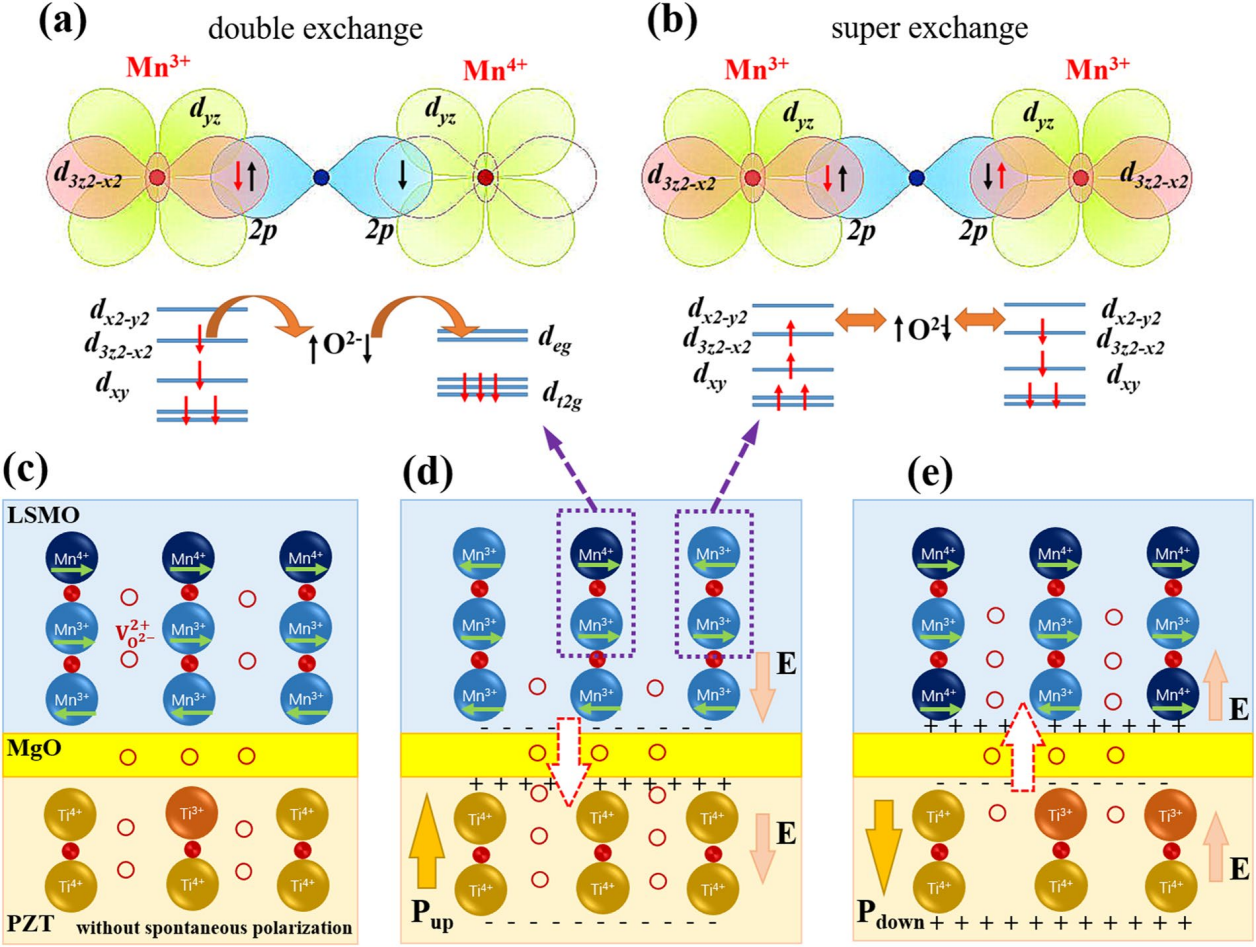
Illustrations of mechanisms of (**a**) double exchange and (**b**) super exchange which lead to the modulation of the change of magnetization, (**c**) motions of oxygen vacancies in the heterostructure without spontaneous polarization, (**d**) upward remnant polarization, and (**e**) downward remnant polarization.

The mechanism of magnetization modulation at the PZT/LSMO interface is illustrated in Fig. 7. It is known that the competition between double exchange (DE) arising from Mn^3+^-O^2−^-Mn^4+^ and superexchange (SE) from Mn^3+^-O^2−^-Mn^3+^ exist in LSMO films^17^. Specifically, the ferromagnetism of LSMO could be enhanced by a DE effect, as shown in Fig. 7(a), while the SE effect on LSMO is expected to decrease the magnetization due to the local antiferromagnetism shown in Fig. 7(b) ^17^. As reported, the oxygen vacancies existed in MgO layer can be controlled by external electric filed^38–40^. Thus, the oxygen vacancies accumulated at the PZT/MgO and MgO/LSMO interfaces can pass through MgO layer with each other. Furthermore, due to the existence of a built-in electric field inside the PZT layer, the oxygen vacancies in the heterostructure could move between PZT and LSMO through the MgO thin layer. Therefore, the movements of oxygen vacancies at the PZT/LSMO interface would modulate the valence of Mn and Ti in the heterostructure, as presented in Eqs (1) and (2)^24, 25^.

Figure 7(c) illustrates the PZT and LSMO layers under an initial state, while the competitions between DE and SE is kept in balance. In Fig. 7(d), the PZT layer is polarized upward, while the built-in electric field at the PZT/MgO/LSMO interface is downward. As seen from Fig. 7(d), the oxygen vacancies could move from LSMO to PZT layers throughout a thin MgO layer. As a result, at the PZT/MgO/LSMO interface, the Mn^4+^ of LSMO layer can be partly transformed to Mn^3+^ while the Ti^3+^ of PZT layer can be partly transformed to Ti^4+^. In the opposite case, as shown in Fig. 7(e), the built-in electric field at the PZT/MgO/LSMO interface would be along upward while the PZT layer is polarized down. Thus oxygen vacancies of the heterostructure can migrate from the PZT layer to the LSMO layer, which leads to the increase of Mn^4+^ at the PZT/MgO/LSMO interface. The magnetization of LSMO can be modulated by changing the ratio of Mn^3+^/Mn^4+^. With decreasing Mn^4+^, the DE effect arisen from Mn^3+^-O^2−^-Mn^4+^ is weakened while the antiferromagnetic SE arising from Mn^3+^-O^2−^-Mn^3+^ becomes stronger^17^, leading to the enhancement of the total magnetization in the heterostructure.

Interestingly, according to the value of ME coupling coefficients in NPL (η = 63%), the modulated thickness in the LSMO layer can be estimated to be about 2.5 nm. Meanwhile, the formation of Ti^3+^ are generated with the changing of Mn^3+^/Mn^4+^ during this processes. As a result, the changes of chemical valence for Mn and Ti at the PZT/LSMO interface may play a dominant role as opposed to external strain or orbital reconstruction, which leads to a high modulation of magnetizations.

## Conclusion

In summary, the multiferroic NPL and NPML heterostructures were epitaxially grown on the conductive NSTO substrates. Interestingly, at room temperature, the non-volatile modulations of magnetic moments up to 63% and 85% were observed inside the NPL and NPML heterostructures, respectively. The motions of oxygen vacancies in the heterostructures play a dominant role in the modulation of magnetization rather than the external strain and orbital reconstruction. With the increase of Mn^4+^, the ferromagnetic DE effect from Mn^3+^-O^2−^-Mn^4+^ is enhanced while the antiferromagnetic SE effect from Mn^3+^-O^2−^-Mn^3+^ is weakened, which leads to the enhancement of the total magnetization of the heterostructures. Our results indicate that the PZT/MgO/LSMO heterostructure is a promising candidate for future high-density non-volatile memories.

## Methods

Stoichiometric Pb_1.1_Zr_0.2_Ti_0.8_O_3_ (PZT), MgO and La_0.7_Sr_0.3_MnO_3_ (LSMO) ceramic targets were prepared by conventional solid reaction methods. Corresponding PZT, MgO and LSMO layers were deposited in sequence on (100) Nb-SrTiO_3_ (NSTO) substrates by pulsed laser deposition with a KrF excimer laser (λ = 248 nm). After deposition, the samples were annealed at 700 °C under an oxygen pressure of 10^4^ Pa for 30 min to reduce the number of oxygen vacancies. The heterostructures of NSTO//PZT/LSMO and NSTO//PZT/MgO/LSMO are henceforth abbreviated as NPL and NPML, respectively. The thickness of the PZT, MgO and LSMO layers were 150 nm, 2.1 nm and 4 nm, respectively. For a comparison, a LSMO single layer with the thickness of 4 nm was deposited on NSTO under the same deposition conditions. X-ray diffraction (XRD) was carried out using a TTRIII multifunction with monochromatic Cu K-alpha radiation. The surface morphology and ferroelectric domain structure were examined by atomic force microscope (AFM, MFP-3D-SA). To characterize the electrical properties of NPL and NPML, patterned Pt electrodes were deposited on the surface of films by dc sputtering. Ferroelectric hysteresis loops were obtained with a TF-Analyzer 1000 at 1 kHz. The magnetic properties were measured by a vibrating sample magnetometer (VSM) option in a physical property measurement system (PPMS-9). X-ray photoelectron spectroscopy (XPS) analyses were measured by using a ESCALAB MK II.
